# HES1-mediated down-regulation of miR-138 sustains NOTCH1 activation and promotes proliferation and invasion in renal cell carcinoma

**DOI:** 10.1186/s13046-023-02625-0

**Published:** 2023-03-28

**Authors:** Shuangjie Liu, Lei Dou, Miao Miao, Xiaojun Man, Baojun Wei, Zhaowei Jiang, Yongze Ouyang, Toshinori Ozaki, Meng Yu, Yuyan Zhu

**Affiliations:** 1 Department of Urology, The First Hospital of China Medical University, Shenyang, 110001 China; 2 Department of Gynecology, The First Hospital of China Medical University, Shenyang, Liaoning 110001 China; 3 Laboratory of DNA Damage Signaling, Chiba Cancer Center Research Institute, Chiba, Japan; 4 Department of Laboratory Animal Science, Key Laboratory of Transgenetic Animal Research. No, China Medical University, 77 Puhe Road, Shenyang North New Area, Shenyang, Liaoning Province 110122 China

**Keywords:** HES1, miR-138–2, NOTCH, Renal cell carcinoma, Proliferation, Invasion

## Abstract

**Background:**

Although the aberrant activation of NOTCH1 pathway causes a malignant progression of renal cell carcinoma (RCC), the precise molecular mechanisms behind the potential action of pro-oncogenic NOTCH1/HES1 axis remain elusive. Here, we examined the role of tumor suppressive miR-138–2 in the regulation of NOTCH1-HES1-mediated promotion of RCC.

**Methods:**

This study employed bioinformatics, xenotransplant mouse models, ChIP assay, luciferase reporter assay, functional experiments, real-time PCR and Western blot analysis to explore the mechanisms of miR-138–2 in the regulation of NOTCH1-HES1-mediated promotion of RCC, and further explored miR-138–2-containing combination treatment strategies.

**Results:**

There existed a positive correlation between down-regulation of miR-138 and the aberrant augmentation of NOTCH1/HES1 regulatory axis. Mechanistically, HES1 directly bound to miR-138–2 promoter region and thereby attenuated the transcription of miR-138-5p as well as miR-138–2-3p. Further analysis revealed that miR-138-5p as well as miR-138–2-3p synergistically impairs pro-oncogenic NOTCH1 pathway through the direct targeting of APH1A, MAML1 and NOTCH1.

**Conclusions:**

Collectively, our current study strongly suggests that miR-138–2 acts as a novel epigenetic regulator of pro-oncogenic NOTCH1 pathway, and that the potential feedback regulatory loop composed of HES1, miR-138–2 and NOTCH1 contributes to the malignant development of RCC. From the clinical point of view, this feedback regulatory loop might be a promising therapeutic target to treat the patients with RCC.

**Supplementary Information:**

The online version contains supplementary material available at 10.1186/s13046-023-02625-0.

## Background

Renal cell carcinoma (RCC) is one of the malignant cancers with an increasing morbidity and mortality in recent years. For example, the global incidence of RCC is over than 72,000 by 2023 [1, 2]. The incidence rate of clear cell renal cell carcinoma (ccRCC) accounts for 75–80% of RCC [3, 4]. Although the targeted therapies such as targeting of VEGFR/PDGFR tyrosine kinases and mTOR pathway, and the immunotherapies (pembrolizumab, ipilumumab, nivolumab) significantly improved the prognosis of metastatic ccRCC [5–7], 30% of RCC patients with metastasis showed a primary resistance to the molecular targeted drugs [6]. Unfortunately, those patients who were initially sensitive to the treatments displayed "secondary drug resistance" around one year after the treatments, which greatly reduces the efficacy of the molecular targeted drugs [8, 9]. To overcome this serious issue, it is urgent to clarify the precise molecular mechanisms behind the progression of RCC and develop a promising therapeutic strategy to treat RCC patients.

NOTCH1-mediated signaling pathway plays a vital role in the regulation of the cellular communication during organ development [10]. Upon the ligand binding, NOTCH1 is subjected to its cleavage, and then its intracellular domain (NICD) forms an active complex with various transcription factors within cell nucleus, thereby transactivating its downstream target genes such as *HES1* (hairy and enhancer of split 1), c-*MYC* and *VCAM1* [11, 12]. Among them, HES1 has been shown to be a transcriptional regulator directly regulated by NOTCH1 and play a pivotal role in down-regulation of NOTCH1-mediated downstream signaling [13, 14]. Of note, accumulating evidence suggests that NOTCH1 has oncogenic role in numerous cancers including RCC [15, 16], breast cancer [17], lung cancer [18], prostate cancer [19] and melanoma [20]. In support of this notion, it has been also described that NOTCH1 requires HES1 to exert its oncogenic function [21, 22]. To date, it still remains unclear how HES1 could participate in NOTCH1-induced development of RCC.

MicroRNA (miRNA) is a small non-coding RNA (18–22 nucleotides in length) [23] which binds to the 3’-untranslated region (3'-UTR) of its target mRNA to prohibit its translation [24]. Its uncontrolled expression is frequently observed in a variety of cancers such as RCC [25], indicating that miRNA might play a crucial role in the regulation of proliferation, differentiation, apoptosis and stress response of cancers [24, 26]. Indeed, it has been described that a variety of miRNAs including miR-19 [27], miR-21 [28] and miR-33a [29] are involved in the regulation of NOTCH1 signaling pathway [30]. Since miRNA might have an ability to attenuate its target gene expression implicated in cancer development, it is suggestive that miRNA is an attractive natural product used for an alternative therapy [31–33]. Thus, the identification of the candidate miRNA(s) participated in NOTCH1-HES1 regulatory complex contributes to the better understanding of NOTCH1-induced RCC development.

In the present study, we have demonstrated that miR-138–2 is NOTCH1-specific microRNA, and its expression is directly down-regulated by HES1. Moreover, our results clearly showed that miR-138 efficiently impairs the translation of major members of NOTCH1 signaling pathway. Collectively, our current findings emphasized the importance of the key regulatory role of HES1-miR-138-NOTCH1 regulatory feedback loop in NOTCH1 signaling pathway, and also strongly suggest that miR-138 is an attractive molecular target for the development of an alternative therapeutic strategy to treat RCC patients.

## Methods

### Cells and cell culture

Human renal cancer-derived 786-O and CAKI-1 cells were purchased from the Chinese Academy of Sciences (Shanghai, China). 786-O and CAKI-1 cells were cultured in RPMI1640 (HyClone) and McCoy's 5A mediums (HyClone), respectively. These mediums were supplemented with 10% heat-inactivated fetal bovine serum (FBS). Cells were maintained at 37 °C in an incubator containing 5% CO_2_.

### Transfection

For transient transfection, the indicated plasmid DNA (5 μg) was introduced into cells in the presence of Lipofectamine 3000 transfection regent (Thermo Fisher) according to the manufacturer’s instructions.

### Patients and clinical samples

From February 2020 to October 2022, a total 60 pairs of clear-cell renal cell carcinoma specimens (tumor tissues and their adjacent healthy ones) were collected at First Hospital of China Medical University (Shenyang, China). All samples were immediately snap-frozen and stored at − 80 °C until required. The Committees for Ethical Review of Research involving Human Subjects at First Hospital of China Medical University provided the ethical consent and the informed consent was signed by all of the patients.

#### EdU assay

Twenty-four hours after transfection, 786-O and CAKI-1 cells were treated with EdU (Sigma) at a final concentration of 10 μM and incubated for 2 h. Then, cells were washed in PBS for 3 times and blocked for 15 min. After blocking, cells were reacted with fluorescent dye mixture (Sigma) for 2 h under the manufacturer’s protocol. Finally, cells were washed in PBS for 3 times, and observed under fluorescence microscope.

#### CCK-8 assay

786-O and CAKI-1 cells were plated in 96-well plates (1,500 cells per well) and transfected with the indicated plasmids. Twenty-four hours after transfection, cells were incubated in a fresh medium containing CCK-8 assay reagent at a final concentration of 0.5 mg/mL (Sigma). One hour after incubation, the absorbance was measured at 450 nm.

#### Migration assay

Cell migration assay was performed using transwell (BD Biosciences). Twenty-four hours after transfection, cells were mixed with 150 μl of serum-free culture medium (1 × 10^4^ cells per chamber) in the upper transwells. Then, 600 μl of serum were added to culture medium in the lower transwells. Twenty-four hours after the incubation, culture medium was replaced by culture medium containing 4% paraformaldehyde and cells were maintained for 20 min. Then, cells were incubated in fresh medium containing crystal violet for 20 min. Finally, cells were washed in PBS, and number of cells was scored.

#### Real-time PCR

Total RNA was prepared from the indicated tissues or cells using Trizol reagent (Invitrogen), and subjected to reverse transcription in the presence of PrimeScript RT Master Mix (Takara). q-PCR was carried out using SYBR premix Ex TaqII reagent (Takara). U6 RNA and β-*Actin* were used as the internal controls. The sequences of primers used in this study were shown in Supplementary Table.

#### Western blot analysis

Membranes were incubated with 5%-10% fat-free milk in TBST. After blocking, membranes were incubated with the indicated primary antibodies at 4 °C overnight. Membranes were washed in TBST for 3 times and mixed with the appropriate secondary antibodies for 1 h at 37 °C. After incubation, membranes were washed in TBST for 3 times, subjected to enhanced chemiluminescence and finally observed under molecular imager ChemiDoc XRS + system. Antibodies used in this study were as follows: anti-HES1 (Abcam), anti-β-Tubulin (Abcam), anti-NOTCH1 (Cell Signaling Technologies), anti-MAML1 (Cell Signaling Technologies), anti-APH1A (Abcam), peroxidase-conjugated goat anti-mouse IgG and peroxidase-conjugated goat anti-rabbit IgG (Abcam).

#### ChIP assay

ChIP assay kit (Millipore) was employed in this study according to the manufacturer’s instructions. In brief, CAKI-1 cells (5 × 10^7^ cells) were immersed in 1% formaldehyde for 12 min, and then incubated in the presence of glycine for 5 min. After incubation, cells were collected, incubated with protease inhibitors and subjected to a brief sonication for 4 min to generate the genomic DNA fragments of around 200 to 500 bp in length. After sonication, anti-HES1 antibody (Santa Cruz Biotechnologies) was added to the reaction mixtures in the presence of protein A–Sepharose beads and the reaction mixtures were incubated at 4 °C. IgG alone was used as a negative control. Twenty-four hours after incubation, the reaction mixtures were rinsed in the indicated washing buffer following the manufacturer’s recommendations. The reaction mixtures were heated at 65 °C for 2 h, the genomic DNA fragments were purified using DNA Spin column (Millipore) and then subjected to q-PCR. qPCR was performed using the following primers: 5′-GGGTCTCTCTTTCCAGGCTGA-3′ (*HES1P1*, forward) and 5′-CCAACCACACAGTGAACCTCA-3′ (*HES1P1*, reverse), or 5′-A TGCTCTTTCCCAGTGTCCG-3′ (*HES1P2*, forward) and 5′-TACCCCGAAGGA GTTTGTGC-3′ (*HES1P2*, reverse).

#### Luciferase reporter assay

The 3′-UTR of *NOTCH1*, *MAML1* and *APH1A* were inserted into PmirGLO dual luciferase miRNA target expression vector to give the wild-type reporter vectors. In addition, the 3′-UTR bearing the mutated miR-138–2-binding site was also inserted into PmirGLO dual luciferase miRNA target expression vector to generate the mutated-type reporter vectors. The indicated cells were transfected with these reporter vectors using Lipo-3000 (Thermo Fisher). Forty-eight hours after transfection, cells were lysed and their luciferase activities were measured.

### Bioinformatic analysis

The RNA-Seq data and the additional patient information were downloaded from the public TCGA (http://cancergenome.nih.gov/) KIRC data repositories. The mutation analysis data focused on NOTCH signaling pathway was downloaded from the data base (http://www.bioinformatics.com.cn). The Kaplan–Meier analysis of TCGA KIRC was downloaded from the data base (https://www.kmplot.com/analysis/). Three microarray data (the accession numbers: GSE16441, GSE37989 and GSE71302) were processed for the GEO analysis. Genes that are significantly correlated with miR-138–2 were determined based on TCGA KIRC data. These selected genes were then subjected to GSEA analysis.

### Mice and treatments

All animals were cared and treated according to the National Institutes of Health guide for the Laboratory animals. Female BALB/c nude mice (4–5 weeks old, weight ~ 20 g) were randomly divided into 2 groups. *HES1*-knocked down, miR-138–2-overexpressing or the control 786-O cells were inoculated in the left groin of mice (5 × 10^6^ cells per mouse). Twenty-four days after the careful observation, mice were weighed and sacrificed. At the same time, xenograft tumors obtained from mice were weighted and measured.

### Statistical analysis

All of the data was analyzed by GraphPad Prism v.9.0. Differences between the indicated groups were calculated using GraphPad Prism Student’s *t*-test. *p* < 0.05 was considered statistically significant.

## Results

### HES1 is involved in NOTCH1-induced clear cell renal cell carcinoma (ccRCC) development

**Fig. 1:**
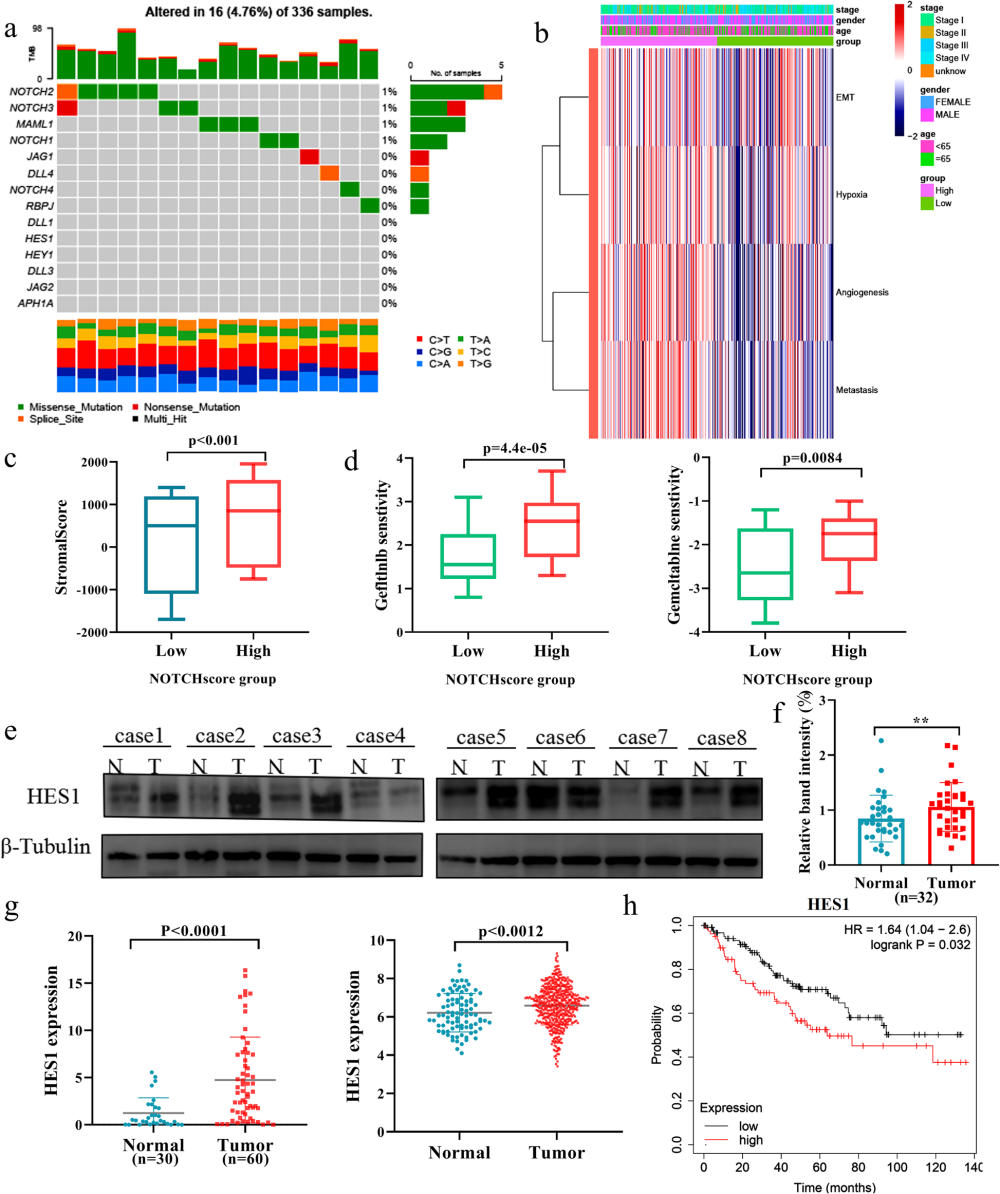
Implication of HES1, a member of NOTCH signaling pathway, in ccRCC. **a** NOTCH signaling pathway-related gene mutations found in TCGA KIRC. **b** Heat map correlation analysis between NOTCH signaling pathway and the malignant features by taking advantage of TCGA KIRC. **c** Relationship between stroma score and NOTCH signaling pathway based on TCGA KIRC. **d** Relationship between gefitinib (left) or gemcitabine (right) sensitivity and NOTCH signaling pathway based on TCGA KIRC. **e** Western blot analysis. Whole cell lysates were prepared from 32 pairs of ccRCC tissues (T) and para-cancerous ones (N), and analyzed for HES1 by western blot. β-tubulin was used as a loading control. **f** Western blot analysis. Relative band intensity analysis of HES1 in 32 cases. **g** qPCR analysis of *HES1* in 60 ccRCC tissues and 30 para-cancerous ones. **h** Expression analysis of *HES1* in TCGA KIRC (left), and Kaplan–Meier analysis based on *HES1* expression in TCGA KIRC cohort (right)

To investigate how NOTCH1 signaling pathway participates in the development of clear cell renal cell carcinoma (ccRCC), at first, we sought to identify the mutated gene(s) related to NOTCH1 signaling pathway based on TCGA KIRC cohort. As shown in Fig. 1a, although several genes involved in NOTCH1 signaling pathway were mutated in ccRCC tissues, the frequency of mutations is low. The results suggest that NOTCH pathway should regulate ccRCC in a non-mutational manner. Subsequently, the heat map analysis revealed that NOTCH1 signaling pathway is significantly associated with the malignant features of ccRCC such as stroma score and chemo-sensitivity (Fig. 1b-d, and Supplementary Fig. 1a). These findings strongly suggest that NOTCH signaling pathway is tightly linked to ccRCC, which was consistent with the previous studies [15, 16]. Among the downstream effectors of NOTCH signaling pathway, we have focused on HES1 with oncogenic potential. To our knowledge, the potential implication of HES1 in ccRCC development has been elusive [13].

To obtain a clue to explore the possible role of HES1 in ccRCC development, we have examined the expression levels of HES1 protein in 32 pairs of ccRCC tissues and their corresponding para-cancerous ones. As clearly seen in Fig. 1e and f, and Supplementary Fig. 1b, HES1 protein was expressed at much higher level in ccRCC tissues relative to their corresponding para-carcinoma ones. To confirm these results, we have examined the expression level of *HES1* mRNA in 60 ccRCC tissues and 30 para-cancerous ones. In accordance with the results obtained from immunoblotting, *HES1* mRNA was highly expressed in ccRCC tissues as compared to para-cancerous ones (Fig. 1g). In support of these observations, HES1 was aberrantly overexpressed in ccRCC tissues relative to para-cancerous ones based on TCGA KIRC (Fig. 1g). Furthermore, Kaplan–Meier analysis based on TCGA KIRC demonstrated that a higher expression level of *HES1* is significantly associated with a poor overall survival of ccRCC patients (Fig. 1h).

### HES1 enhances cellular proliferation and migration abilities of ccRCC-derived cells

To examine whether HES1 could enhance cellular proliferation and migration abilities of ccRCC, *HES1* gene silencing was performed in ccRCC-derived 786-O and CAKI-1 cells. Under our experimental conditions, the endogenous HES1 was successfully down-regulated in 786-O and CAKI-1 cells (Supplementary Fig. 1c). In addition, we designed a rescue experiment to verify the specificity of HES1 bands in western blot (Supplementary Fig. 1d). To check the possible effect of *HES1* depletion on their migration and proliferation abilities, knockdown cells were subjected to transwell and CCK-8 assays, respectively. As shown in Supplementary Fig. 1e and f, number of the migrated cells was obviously decreased in response to *HES1* depletion. Consistent with these observations, CCK-8 assay revealed that the proliferation rate of *HES1*-knockdown cells markedly reduces as compared to the control cells (Supplementary Fig. 1 g). In addition, EdU assay showed that gene silencing of *HES1* causes a significant decrease in number of EdU-positive cells relative to the control transfection (Supplementary Fig. 1 h-i). Together, our present results strongly indicate that HES1 has an ability to promote cellular proliferation and migration of ccRCC cells.

### miR-138–2 is tightly linked to NOTCH pathway and to a better prognosis of patients with ccRCC

**Fig. 2:**
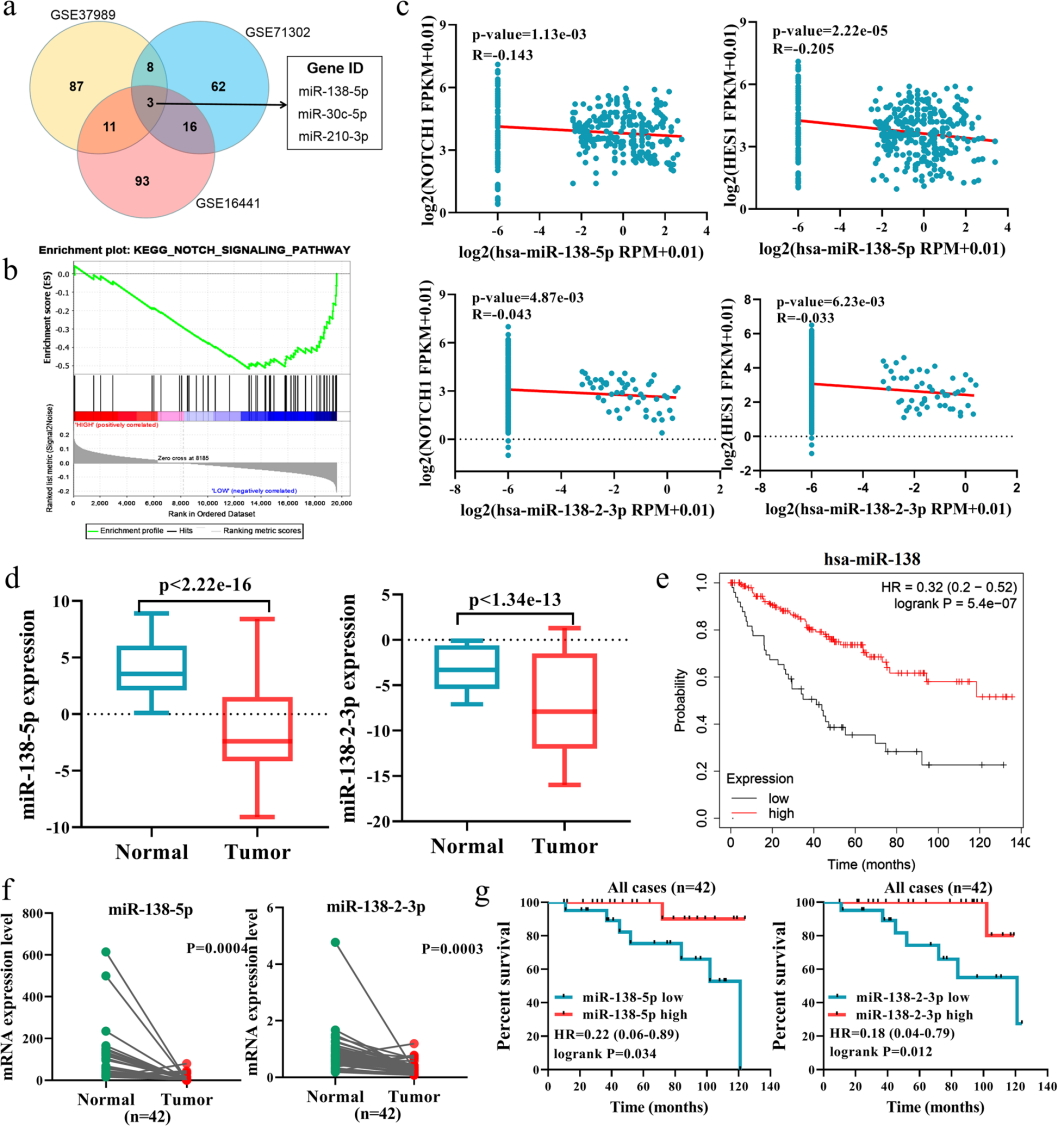
Negative correlation between miR-138–2 and Notch signaling pathway in renal cell carcinoma. **a** Identification of the differentially expressed microRNAs by taking advantage of 3 independent cohorts (GSE16441, GSE37989, and GSE71302). **b** GSEA analysis of the possible relationship between miR-138–2 and NOTCH signaling pathway based on TCGA KIRC cohort. **c** Inverse relationship between miR-138-5p/miR-138–2-3p and the members of NOTCH signaling pathway genes (NOTCH1 and HES1) in TCGA KIRC samples. **d** Expression analysis of miR-138-5p and miR-138–2-3p in TCGA cohort. **e** Kaplan–Meier analysis of the patients with ccRCC based on miR-138 expression level obtained from TCGA KIRC cohort. **f** qPCR analysis of miR-138-5p and miR-138–2-3p in 42 pairs of RCC tissues and their adjacent normal kidney ones. **g** Kaplan–Meier analysis based on miR-138-5p and miR-138–2-3p expression level in 42 pairs of ccRCC tissues and para-cancerous ones. The cut-off for Kaplan–Meier analysis was according to median mRNA expression level

Since miRNAs have been shown to be frequently dysregulated in cancer, we sought to identify miRNA(s) dysregulated in ccRCC. To this end, we utilized the miRNA datasets in GEO: GSE144082, GSE95385, and GSE116251. As shown in Fig. 2a, several miRNAs were clearly down-regulated in ccRCC tissues relative to para-carcinoma ones. To find out miRNA(s) related to NOTCH signaling pathway among them, we performed gene set enrichment analysis (GSEA) to examine the expression level of miRNAs based on TCGA KIRC. As seen in Fig. 2b, miR-138–2 was significantly related to NOTCH signaling pathway. Since it has been well-known that the primary miRNA is cleaved by Drosha and Dicer enzymes, and then the mature miRNA is generated, we have checked the expression levels of the mature miRNAs such as miR-138-5p and miR-138–2-3p. As shown in Fig. 2c, miR-138-5p and miR-138–2-3p were inversely associated with the expression levels of *NOTCH1* and *HES1*. Based on these results, it is suggestive that miR-138–2 is related to NOTCH signaling pathway to a higher degree.

To address the clinical significance of miR-138–2 in ccRCC, we have examined the expression levels of miR-138-5p and miR-138–2-3p based on TCGA KIRC cohort. As seen in Fig. 2d, miR-138-5p and miR-138–2-3p were expressed at a higher level in normal renal tissues as compare to ccRCC ones. Consistent with these results, the Kaplan–Meier analysis based on TCGA KIRC cohort revealed that a higher expression level of miR-138–2 is closely associated with a better prognosis of the patients bearing ccRCC (Fig. 2e). In addition, we have compared the expression levels of miR-138-5p and miR-138–2-3p between the paired ccRCC tissues and para-cancerous ones (*n* = 42) (Fig. 2f), and carried out Kaplan–Meier analysis based on these results (Fig. 2g). Collectively, our present results indicate that miR-138–2 is related to a favorable prognosis of the patients with ccRCC.

### HES1 prohibits miR-138–2-mediated suppression of cellular proliferation and migration of ccRCC cells

**Fig. 3:**
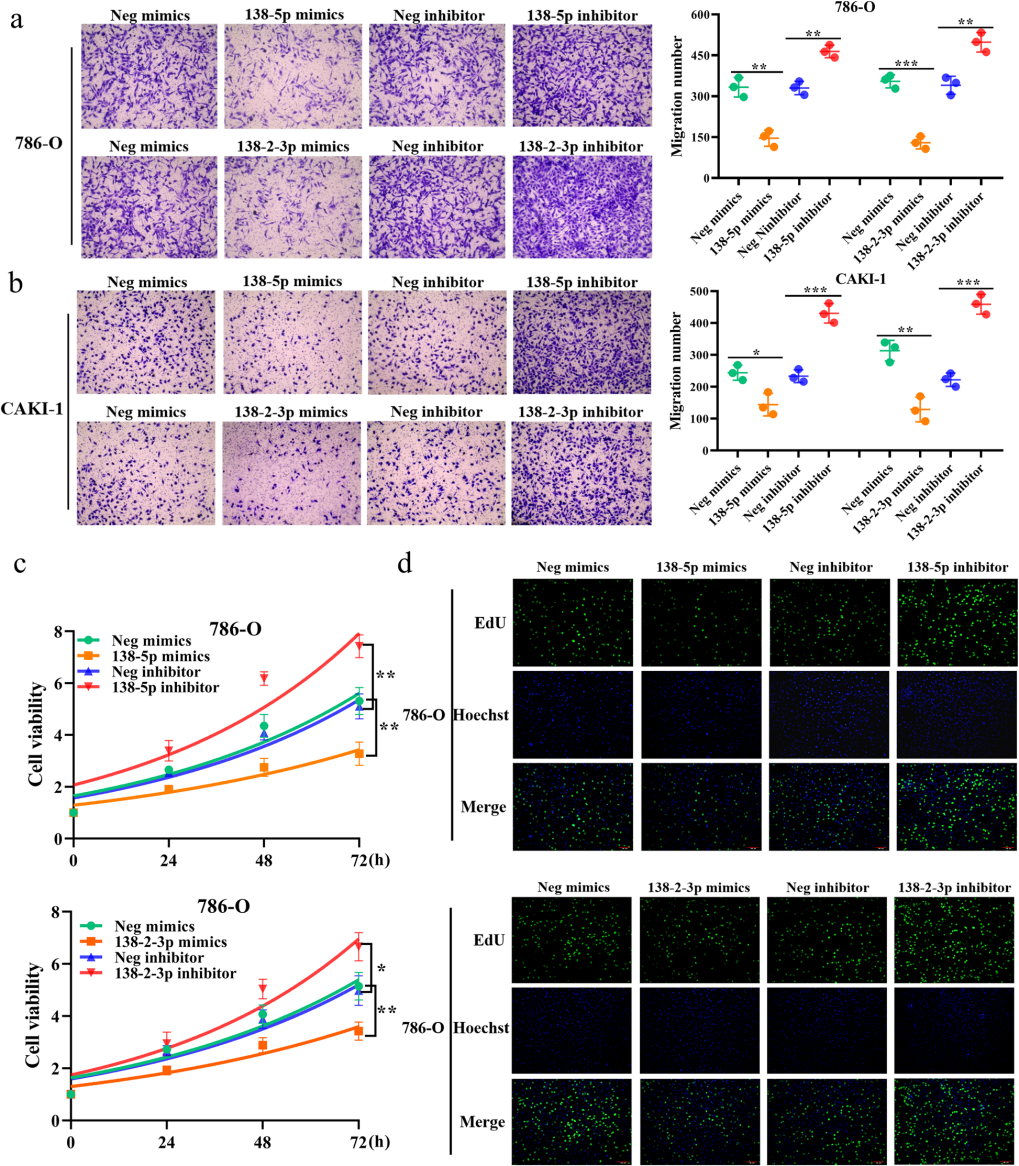
miR-138-5p and miR-138–2-3p reduce cellular proliferation and migration abilities of ccRCC cells. **a** Transwell assay. 786-O cells were transfected with miR-138-5p mimics, miR-138-5p inhibitor or with the appropriate negative controls. After transfection, their migration ability was examined by transwell assay (left). Number of the migrated cells were scored (right). Similarly, 786-O cells were transfected with miR-138–2-3p mimics, miR-138–2-3p inhibitor or with the appropriate negative controls. After transfection, their migration ability was examined by transwell assay (left). Number of the migrated cells were scored (right). **b** Transwell assay. CAK-1 cells were transfected as in **a**. After transfection, their migration ability was examined by transwell assay (left). Number of the migrated cells were scored (right) (*n* = 3). **c** CCK-8 assay. 786-O cells were transfected as in (**a**). At the indicated time points after transfection, cell viability was examined by CCK-8 assay (*n* = 3). (d) EdU assay. 786-O cells were transfected as in (**a**). After transfection, cells were subjected to EdU assay. Cell nuclei were stained with Hoechst

To elucidate the possible role of miR-138–2 in the regulation of cellular proliferation and migration in ccRCC, ccRCC-derived 786-O and CAKI-1 cells were transfected with miR-138-5p mimics, its inhibitor, miR-138–2-3p mimics or with its inhibitor (Supplementary Fig. 2a and b). As shown in Fig. 3a and b, forced expression of miR-138-5p or miR-138–2-3p markedly reduced the migration rate of 786-O and CAKI-1 cells. By contrast, 786-O and CAKI-1 cells treated with the inhibitor against miR-138-5p or miR-138–2-3p displayed the increased migration rate. In accordance with these observations, their proliferation rates were decreased and increased in the presence of the exogenous miR-138-5p or miR-138–2-3p and the inhibitor against miR-138-5p or miR-138–2-3p, respectively (Fig. 3c-d and Supplementary Fig. 3a-d). Thus, our observations strongly indicate that miR-138–2 is implicated in the regulation of ccRCC proliferation and migration.

**Fig. 4:**
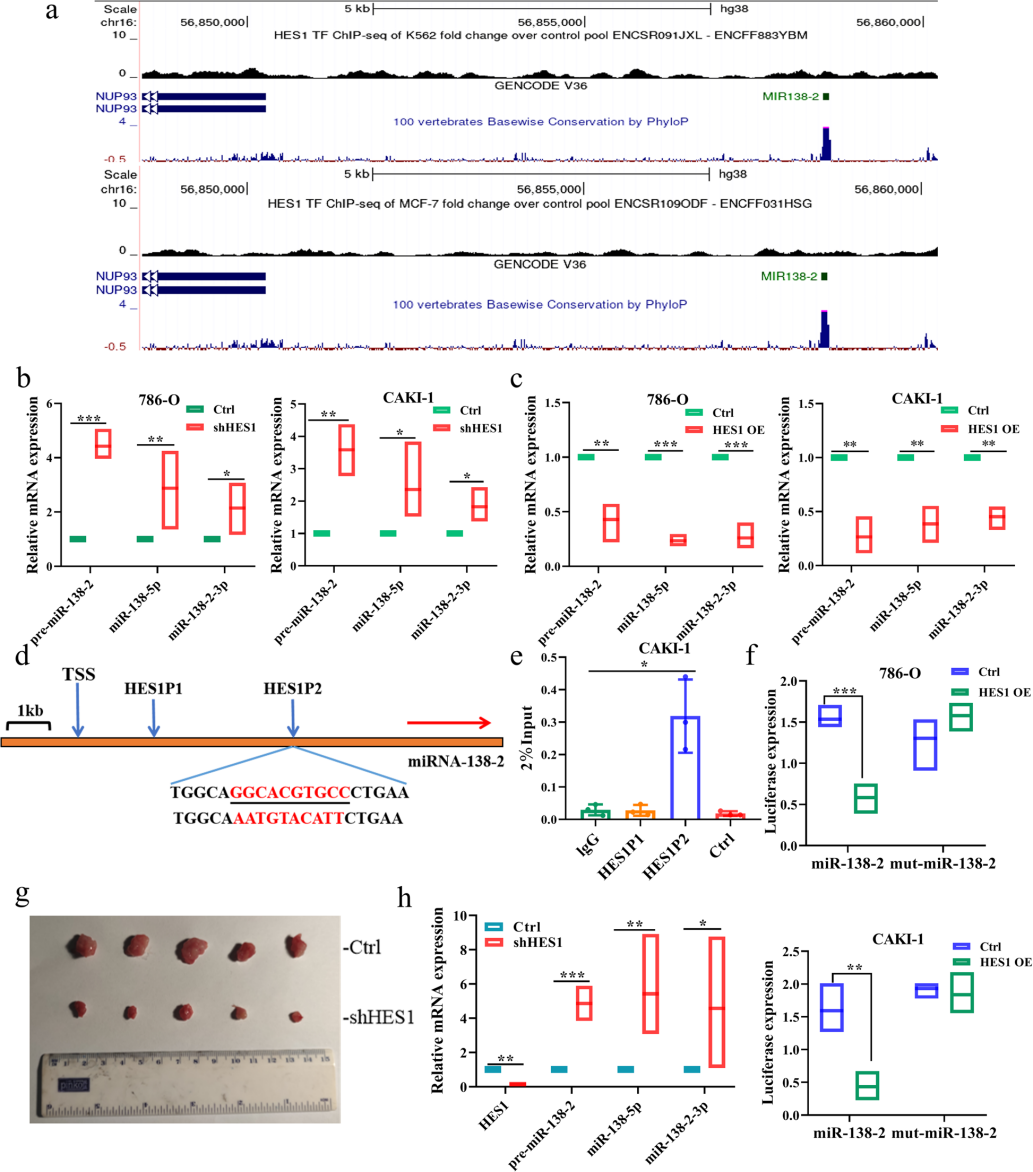
HES1-mediated down-regulation of miR-138–2 in ccRCC cells. **a** The putative HES1-binding site within the promoter region of miR-138–2. ChIP-seq data of K562 and MCF-7 cells obtained from the ENCODE project was shown. **b** Knockdown of *HES1*. 786-O (left) and CAKI-1 (right) cells were transfected with the control shRNA or with shRNA against *HES1*. After transfection, total RNA was prepared and analyzed for pre-miR-138–2, miR-138-5p and miR-138–2-3p by qPCR (*n* = 3). **c** Overexpression of HES1. 786-O (left) and CAKI-1 (right) cells were transfected with the empty plasmid or with the expression plasmid for HES1. After transfection, total RNA was prepared and analyzed for pre-miR-138–2, miR-138-5p and miR-138–2-3p by qPCR (*n* = 3). **d** Schematic diagram of the predicted HES1-binding sites within miR-138–2 genomic locus. **e** ChIP assay. Cross-linked CAK-1 genomic DNA was immunoprecipitated with the control IgG or with anti-HES1 antibody. The fragmented genomic DNA was purified from the immunoprecipitates and then subjected to qPCR with HES1P1- or with HES1P2-specific primers (*n* = 3). **f** Luciferase reporter assay. 786-O (upper) or CAK-1 (lower) cells were transfected with the luciferase reporter construct bearing 4-kb upstream sequence of miR-138–2 or its mutated sequence together with or without HES1 expression plasmid. After transfection, cells were lysed and their luciferase activities were measured (*n* = 3). **g** Mouse xenograft. Nude mice were injected with the control 786-O cells or with *HES1*-depleted 786-O cells. The representative pictures of the indicated tumors were shown. **h** qPCR. Total RNA was prepared from the indicated tumor tissues and analyzed for pre-miR-138–2, miR-138-5p and miR-138–2-3p by qPCR (*n* = 5)

Upon the close inspection of the HES1 ChIPseq data of the ENCODE project, we found out the strong HES1-binding sites within the promoter region of miR-138–2 as examined in K562 and MCF-7 cells (Fig. 4a). To address whether there could exist the functional interplay between miR-138–2 and HES1, shRNA-mediated knockdown of *HES1* was performed in 786-O and CAKI-1 cells. As shown in Fig. 4b, depletion of *HES1* induced pre-miR-138–2, miR-138-5p and miR-138–2-3p. In support of these results, forced expression of HES1 reduced the expression levels of pre-miR-138–2, miR-138-5p and miR-138–2-3p (Fig. 4c). Of note, the analysis of miR-138–2 genomic locus by taking advantage of Encode website revealed that two putative HES1-binding sites (HESP1 and HESP2) exist at 6-kb and 4-kb upstream relative to miR-138–2 transcriptional start site (Fig. 4d). To ask whether HESP1 and HESP2 could be functional under our experimental conditions, we carried out ChIP assay. As shown in Fig. 4e, HESP2 but not HESP1 was co-precipitated with HES1. As expected, our luciferase reporter assay demonstrated that HES1 has an ability to drive miR-138–2 promoter bearing wild-type HESP2 but not miR-138–2 promoter carrying mutated HESP2 (Fig. 4f). Therefore, it is suggestive that HES1 directly binds to HESP2 of miR-138–2. Consistent with our results, a marked up-regulation of pre-miR-138–2, miR-138-5p and miR-138–2-3p was detectable in *HES1*-depleted tumors (Fig. 4g and h), and the tumor volume and weight were obviously reduced in *HES1*-depleted tumors (Supplementary Fig. 4a and b). In addition, the volume and weight of tumors grew faster when *miR-138–2* was knocked down along with *HES1*, compared with *HES1* alone (Supplementary Fig. 4c-e). This further confirmed that HES1 promoted the malignant development of ccRCC by inhibiting the transcription of miR-138–2.

### miR-138–2 participates in the feedback regulatory loop of HES1 regulating NOTCH1, MAML1 and APH1A

**Fig. 5:**
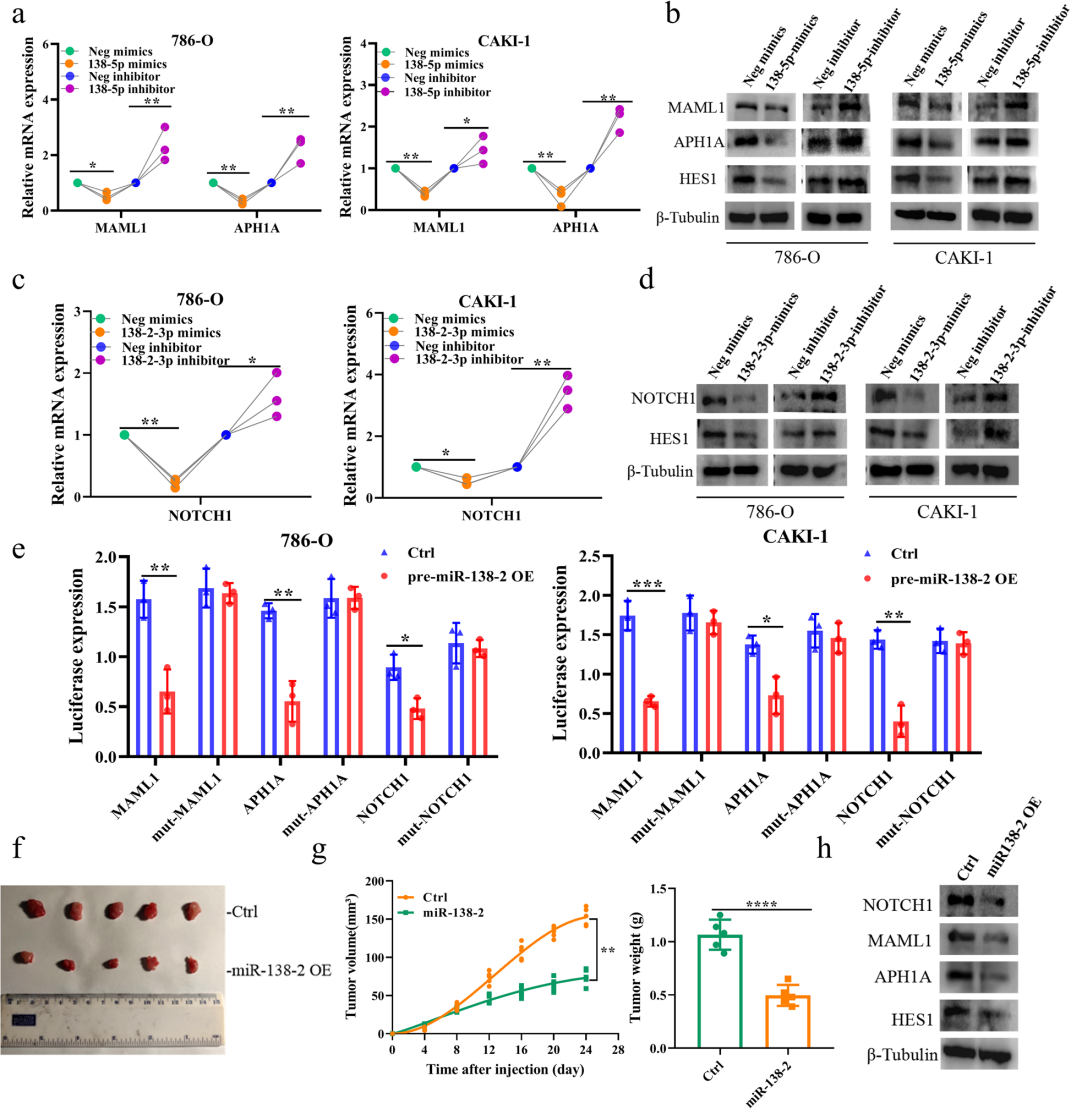
miR-138-2p directly regulates MAML1, APH1A and NOTCH1 in ccRCC cells. **a** and **b** Regulation of MAML1 and APH1A by miR-138-5p. 786-O and CAKI-1 cells were transfected with miR-138-5p mimics, inhibitor or with their corresponding negative controls. After transfection, total RNA and whole cell lysates were prepared and analyzed by qPCR (*n* = 3) (**a**) and immunoblotting (**b**), respectively. β-tubulin was used as a loading control. **c** and **d** Regulation of NOTCH1 by miR-138–2-3p. 786-O and CAKI-1 cells were transfected with miR-138-2p mimics, inhibitor or with their corresponding negative controls. After transfection, total RNA and whole cell lysates were prepared and analyzed by qPCR (*n* = 3) (**c**) and immunoblotting (**d**), respectively. β-tubulin was used as a loading control. **e** Luciferase reporter assay. 786-O (left) and CAK-1 (right) cells were transfected with the luciferase reporters bearing wild-type or mutated 3’-UTR of *MAML1*, *APH1A* or *NOTCH1* together with the control expression plasmid or with pre-miR-138–2 expression plasmid. After transfection, cells were lysed and their luciferase activities were measured (*n* = 3). **f** and **g** Mouse Xenograft. Nude mice were injected with the parental 786-O (upper) or with 786-O cells stably overexpressing miR-138–2 (lower). The representative tumors were shown (**f**). Tumor volume and weight were also calculated (*n* = 5) (**g**). **h** Immunoblotting. Whole cell lysates were prepared from the tumor tissues and analyzed for the indicated proteins. β-tubulin was used as a loading control

Since there existed a strong relationship between miR-138–2 and NOTCH signaling pathway, we sought to examine the functional implication of miR-138–2 in NOTCH signaling pathway. By adding exogenous miR-138-5p or miR-138–2-3p, we found that only MAML1, APH1A and NOTCH1 mRNA levels decreased significantly in the NOTCH pathway (Supplementary Fig. 5a). Based on Targetscan website investigation, we found that MAML1 and APH1A are down-regulated in the presence of the exogenous miR-138-5p at mRNA and protein levels (Fig. 5a and b), and NOTCH1 is also decreased in the presence of the exogenous miR-138–2-3p (Fig. 5c and d). In accordance with these results, miR-138–2 reduced the luciferase activity driven by wild-type 3’-UTR of *MAML1*, *APH1A* or *NOTCH1* reporter, whereas had an undetectable effect on the mutated 3’-UTR of *MAML1*, *APH1A* or *NOTCH1* reporter (Fig. 5e and Supplementary Fig. 5b). Indeed, forced expression of miR-138–2 suppressed the tumor growth in vivo along with a significant down-regulation of NOTCH1, MAML1, APH1A and HES1 (Fig. 5f-h and Supplementary Fig. 5c).

### DBZ, an inhibitor against NOTCH signaling pathway, enhances miR-138–2 and thereby prohibits cellular proliferation as well as migration of ccRCC cells

**Fig. 6:**
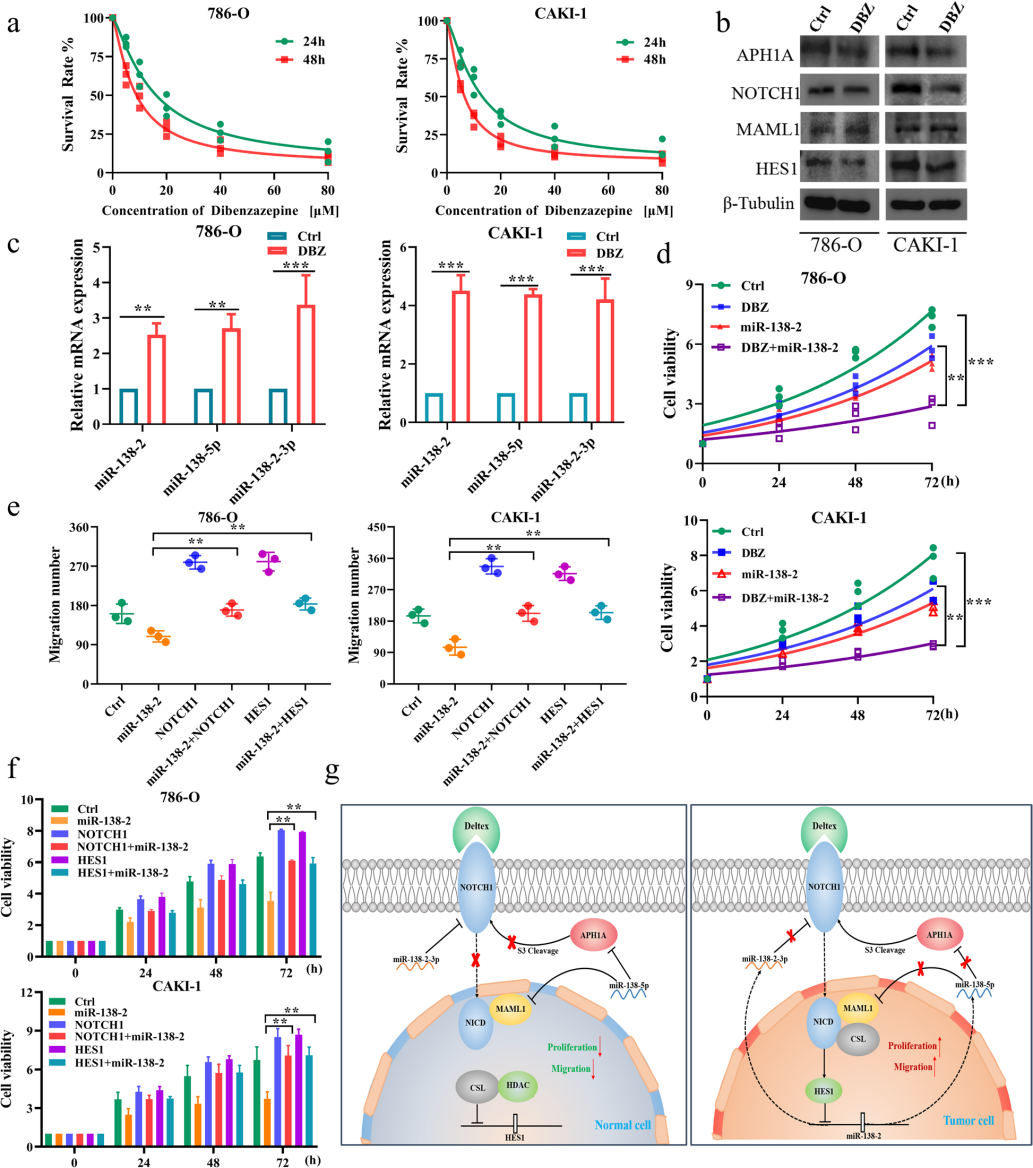
MiR-138–2 enhances DBZ-mediated inhibition of Notch pathway. **a** CCK-8 assay. 786–0 (left) and CAKI-1 (right) cells were exposed to the indicated concentrations of DBZ. At the indicated time points after treatment, cell viability was examined by CCK-8 assay (*n* = 3). **b** Immunoblotting. 786–0 (left) and CAKI-1 (right) cells were treated with the vehicle or with DBZ (at a final concentration of 10 μM/ml). After treatment, whole cell lysates were prepared and analyzed by immunoblotting. β-tubulin was used as a loading control. **c** qPCR. 786–0 (left) and CAKI-1 (right) cells were treated as in (**b**). After treatment, total RNA was isolated and analyzed for pre-miR-138–2, miR-138-5p and miR-138–2-3p by qPCR (*n* = 3). **d** CCK-8 assay. 786–0 (upper) and CAKI-1 (lower) cells were transfected with or without miR-138–2 expression plasmid. After transfection, cells were treated with DBZ (5 μM/ml) or left untreated. At the indicated time periods after treatment, cell viability was assessed by CCK-8 assay (*n* = 3). **e** Migration assay. 786–0 (left) and CAKI-1 (right) cells stably overexpressing NOTCH1 or HES1 were transiently transfected with or without miR-138–2 expression plasmid. Cells were then subjected to transwell assay (*n* = 3). **f** CCK-8 assay. 786–0 (left) and CAKI-1 (right) cells stably overexpressing NOTCH1 or HES1 were treated as in (**e**). At the indicated time points after transfection, cell viability was examined by CCK-8 assay (*n* = 3). **g** Proposed model for miR-138–2 modulates the NOTCH signaling pathway in Normal cell (left) and HES1-miR-138–2-NOTCH1 positive feedback loop in Tumor cell (right). In normal cells, the mature miR-138-5p and miR-138–2-3p inhibited the mRNA expression of MAML1, APH1AH and NOTCH1, respectively, to prevent excessive cell proliferation and migration. However, in tumor cells, over-activated HES1 transcription inhibits *miR-138–2* gene, enabling tumor to activate the NOTCH1-HES1 axis without control, promoting tumor proliferation and metastasis

It has been well-known that dibenzazepine (DBZ), a classical inhibitor against NOTCH signaling pathway, has an ability to prohibit NICD transcription complex [34]. To ask the possible effect of DBZ on ccRCC cells, 786-O and CAKI-1 cells were exposed to the indicated concentrations of DBZ. As shown in Fig. 6a, the viability of these cells was decreased in a concentration-dependent manner. Then, whole cell lysates were prepared from these cells treated with DBZ (10 μM/L) for 24 h, and analyzed for NOTCH1, MAML1, APH1A and HES1 by immunoblotting. As shown in Fig. 6b, NOTCH signaling pathway-related protein expression was obviously attenuated by DBZ. By contrast, the expression level of pre-miR-138–2, miR-138-5p and miR-138–2-3p was elevated in cells exposed to DBZ (Fig. 6c). Next, we have examined the possible effect of DBZ in combination with miR-138–2 on cellular viability. For this purpose, 786-O and CAKI-1 cells were transfected with the control plasmid or with miR-138–2 plasmid, and then exposed to 5 μM/ml of DBZ or left untreated. As shown in Fig. 6d, overexpression of miR-138–2 enhanced the negative-effect of DBZ on cellular viability. Moreover, we designed a rescue experiment to inhibit the expression of miR-138-5p or miR-38–2-3p while treating cells with DBZ. Our results further reveled that DBZ could inhibit cell viability by activating the expression of miR-138–2 (Supplementary Fig. 6a).

To further confirm whether miR-138–2 could target NOTCH1-HES1 regulatory axis, 786-O and CAKI-1 cells were transfected with the indicated combinations of the expression plasmids. As shown in Fig. 6e and f, forced expression of miR-138–2 attenuated their cellular proliferation and migration, whereas NOTCH1 or HES1 impeded the effect of miR-138–2. Taken together, it is likely that HES1 plays an oncogenic role in ccRCC development by directly targeting miR-138–2, and miR-138–2 acts as a tumor-suppressor in ccRCC development through the direct targeting of the oncogenic NOTCH signaling pathway.

## Discussion

Accumulating evidence suggests that NOTCH signaling pathway is augmented in a variety of cancers including glioblastoma, prostate cancer, breast cancer, colorectal cancer and renal cancer [35]. NOTCH1 with an oncogenic potential, participates in various cancer biology such as a maintenance of cancer stem cell-like cells, immunity and angiogenesis [36]. Among the downstream targets of NOTCH1, HES1 has been shown to regulate the key oncogenic pathways, including PI3K and NF-κβ signaling [37, 38]. To our knowledge, it has been elusive how miRNA could participate in the regulation of NOTCH1-HES1-mediated promotion of cancer.

In the present study, we have found a novel NOTCH1-HES1 regulatory axis, which is implicated in the development of ccRCC. Based on our present results, the activation of this regulatory axis enhanced the cellular proliferation and migration of ccRCC cells. Of note, our results demonstrated that miR-138–2 prohibits the malignant phenotypes of ccRCC cells, indicating that miR-138–2 contributes to the efficient inactivation of NOTCH1-HES1 signaling pathway.

It has been well-established that NOTCH/HES1 regulatory axis plays a pivotal role in the malignant progression of the solid and hematological tumors [22]. HES1 is one of the targets of NOTCH signaling, which regulates a variety of key genes implicated in NOTCH signaling pathway. Since HES1 stands at the crossroads of the multiple signaling pathways and is tightly related to tumor outcomes, it is likely that HES1 is a potential therapeutic target. At present, a variety of miRNA have been found to regulate the expression of the members of NOTCH signaling pathway, and the double negative feedback loop formed between HES1 and miR-9 might regulate the proliferation and differentiation of neural progenitor cells [39]. These findings prompted us to ask whether miRNA could modulate NOTCH/HES1 regulatory axis in ccRCC. According to our present results, miR-138–2 was down-regulated in response to the activation of HES1, indicating that there exists a functional interaction between HES1 and miR-138–2 in ccRCC cells. Consistent with these observations, HES1-mediated reduction of miR-138–2 caused a significant increase in the proliferation and migration rates of ccRCC cells. More importantly, our results clearly demonstrated the direct transcriptional inhibition of miR-138–2 by HES1. These findings imply for the first time that non-coding RNA acts as a direct downstream effector of NOTCH1-HES1 in solid tumors. The extensive examination of non-coding RNAs and their regulatory networks might contribute to the better understanding of NOTCH pathway-mediated cancer development.

Another finding of this study was that miR-138–2 has an inhibitory effect on NOTCH1 signaling pathway. It has been described that miR-138 is down-regulated in numerous malignant tumors [40], and regulates the migration, invasion and proliferation of a variety of solid tumors such as colorectal cancer, gastric cancer, cervical cancer, liver cancer, and renal cell carcinoma [40–42]. Of note, various studies have demonstrated that miR-138 has a tumor-suppressive role through the targeting certain oncogenes. In this study, we confirmed that both miR-138-5p and miR-138–2-3p, the mature bodies of miR-138–2, are specifically inhibited in ccRCC, and that high expression of both is associated with benign prognosis of patients. Surprisingly, we found that miR-138–2-3p had the same inhibitory effect on ccRCC as miR-138-5p, indicating that miR-138–2-3p could also be used as a detection indicator of ccRCC to judge the malignancy of tumors. In the present study, we have identified the novel miR-138-5p targets related to NOTCH signaling pathway such as APH1A and MAML1. APH1A, a non-catalytic subunit of the γ-secretase complex, which is responsible for the intramembrane cleavage of Notch receptors [10]. MAML1 is one of the core activation partners to recognize NICD/CSL interface and acts as a transcriptional coactivator of NOTCH signaling pathway [43]. At present, Notch targeting therapies by taking advantage of γ-secretase modulator (GSI) and MAML1 are under development. Based on the current results, we have found for the first time that miR-138 effectively attenuates oncogenic NOTCH signaling pathway in ccRCC cells. It is suggestive that, in addition to the previously identified mechanisms by which miRNA regulates the expression of NOTCH ligands, the receptors and the pathway-related proteins, our present findings postulate a novel mechanism by which miR-138–2 efficiently inhibits the activation of NOTCH signaling pathway.

Although various inhibitors against NOTCH signaling pathway have initially shown the potential therapeutic efficacy in many preclinical models, the treatment effect has been still unsatisfactory. To develop a novel NOTCH targeted therapy, it is urgent to understand the regulatory mechanisms of NOTCH signaling pathway in specific cancers such as ccRCC. Our present findings demonstrated that forced expression of miR-138–2 markedly suppress the malignant phenotypes of ccRCC cells with higher NOTCH1. In addition, miR-138–2 alone or in combination with NOTCH inhibitor DBZ had an inhibitory effect on ccRCC cells. Considering that the targeted miRNA strategy gives us the innovative treatment opportunities, our current results provide a preclinical evidence showing that NOTCH1/HES1 signaling pathway is an alternative target to suppress ccRCC, and that miR-138–2 has an ability to attenuate NOTCH1/HES1 signaling pathway.

## Conclusions

In conclusion, we have found for the first time that HES1-mediateed down-regulation of miR-138–2 augments oncogenic NOTCH1 signaling pathway. Monitoring the status of HES1/miR-138–2/NOTCH1 regulatory axis contributes to the accurate prediction of the risk of the disease recurrence, and miR-138–2 is a promising candidate for the development of a novel therapeutic strategy.

## Supplementary Information

**Additional file 1.**

**Additional file 2.**

**Additional file 3.**

## Data Availability

The data in the current study are available from the corresponding author upon request.

## Abbreviations

RCC: Renal cell carcinoma
ccRCC: Clear cell renal cell carcinoma
NICD: NOTCH1 intracellular domain
miRNA: MicroRNA
3'-UTR: 3’-Untranslated region
GSEA: Gene set enrichment analysis
DBZ: Dibenzazepine
TGCA: Cancer genome atlas program
