# Supplementary material for: HES1-mediated down-regulation of miR-138 sustains NOTCH1 activation and promotes proliferation and invasion in renal cell carcinoma
Source: J Exp Clin Cancer Res. 2023 Mar 28;42:72. doi: 10.1186/s13046-023-02625-0 (PMC10045948; doi:10.1186/s13046-023-02625-0)
Supplement: Supplementary file 1 — Additional file 1. [file 13046_2023_2625_MOESM1_ESM.doc]

**Supplementary Table 1. Correlation between expression of miR-138-5p, miR-138-2-3p and clinicopathological parameters in 42 cases of ccRCC patients**

|  |  | miR-138-5p |  | miR-138-2-3p |  |  |
| --- | --- | --- | --- | --- | --- | --- |
| VariablesGroup | N | LOW HIGH | *P* valus | LOW HIGH | *P* valus |  |
| Gender |  |  |  | 0.349 |  | 0.118 |
|  | Male | 24 | 14 10 |  | 15 9 |  |
|  | Femal | 18 | 7 11 |  | 6 12 |  |
| Age |  |  |  | 0.058 |  | 0.011* |
|  | ≤60 | 25 | 16 9 |  | 17 8 |  |
|  | >60 | 17 | 5 12 |  | 4 13 |  |
| T classification |  |  |  | 0.009* |  | 0.067 |
|  | T1/T2 | 32 | 12 20 |  | 13 19 |  |
|  | T3/T4 | 10 | 9 1 |  | 8 2 |  |
| Distant metastasis |  |  |  | 0.999 |  | 0.232 |
|  | Negative | 39 | 20 19 |  | 18 21 |  |
|  | Positive | 3 | 1 2 |  | 3 0 |  |
| Lymphatic invasion |  |  |  |  |  | - |
|  | Positive | 0 | - - |  | - - |  |
