# Supplementary material for: HES1-mediated down-regulation of miR-138 sustains NOTCH1 activation and promotes proliferation and invasion in renal cell carcinoma
Source: J Exp Clin Cancer Res. 2023 Mar 28;42:72. doi: 10.1186/s13046-023-02625-0 (PMC10045948; doi:10.1186/s13046-023-02625-0)
Supplement: Supplementary file 2 — Additional file 2. [file 13046_2023_2625_MOESM2_ESM.doc]

**Supplement Figures**


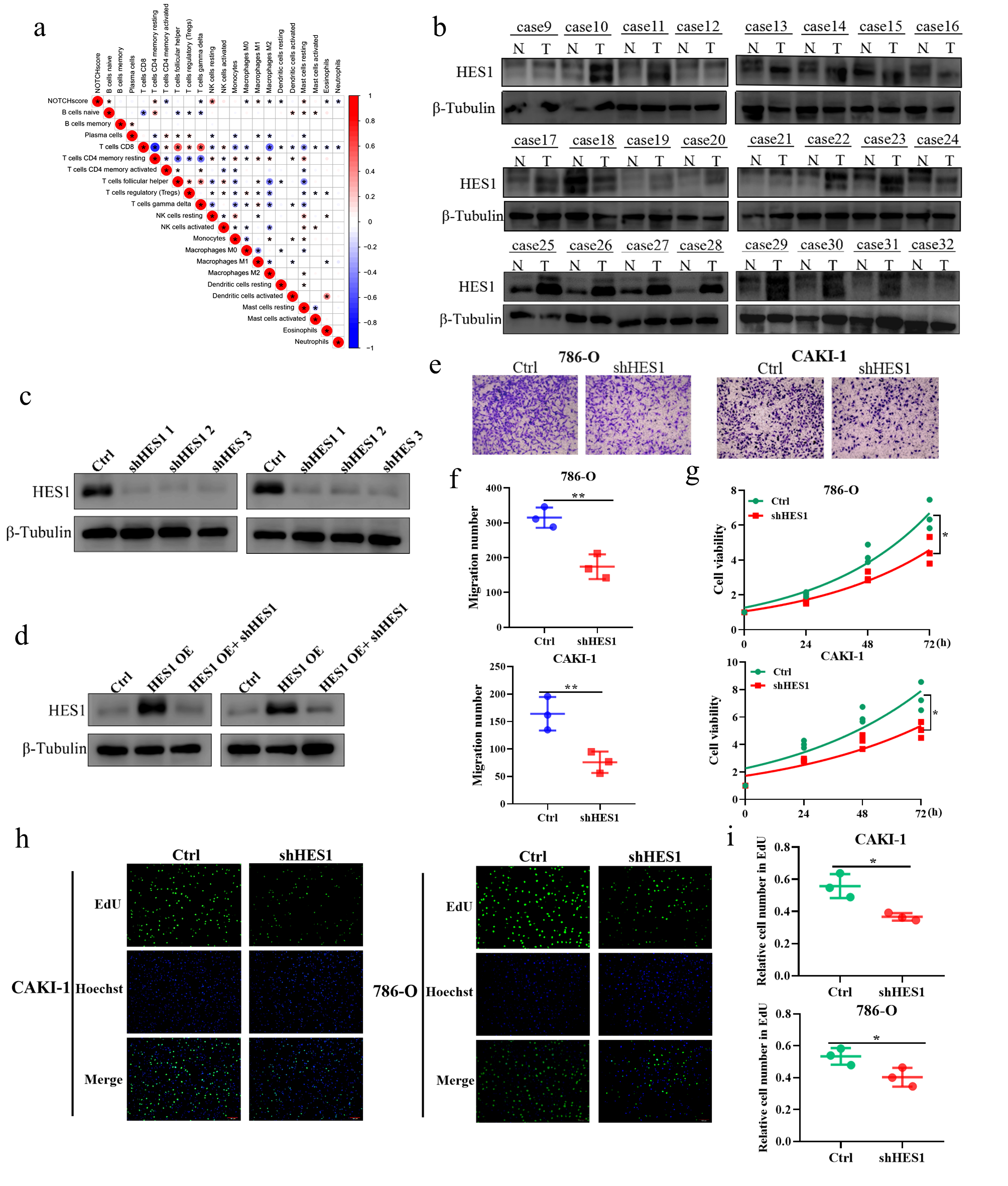


**Supplementary Figure S1. Depletion of HES1 reduces the migration and proliferation ability of ccRCC cells.** (a) Correlation between NOTCH signaling pathway and the indicated immune cells in TCGA KIRC. (b) Higher expression of HES1 in RCC tumor tissues. Whole cell lysates were prepared from 32 pairs of RCC tissues and their adjacent normal kidney ones, and analyzed for HES1 by immunoblotting. β-tubulin was used as a loading control. (c) Knockdown of HES1. 786-O (left panels) and CAKI-1 (right panels) cells were transfected with the scrambled shRNA (Ctrl) or with shRNA against HES1. The amount of HES1 was examined by Western blot. β-tubulin was used as a loading control. (d) Western blot analysis. A rescue experiment to verify the specificity of HES1 bands in western blot. 786-0 (left) and CAKI-1 (right) cells stably overexpressing HES1 were transiently transfected with or without miR-138-2 expression plasmid. The amount of HES1 was examined by Western blot. β-tubulin was used as a loading control. (e and f) Transwell assays. The control and HES1-knocked down 786-0 (upper panels) or CAKI-1 (lower panels) cells were subjected to transwell migration assay. The migrated cells were stained by crystal violet (d). Number of the migrated cells was scored (n=3) (f). (g-i) Proliferation assay. 786-0 (right panels) or CAKI-1 (left panels) cells were transfected as in (d). At the indicated time periods after transfection, cells were processed for CCK-8 (n=3) (g) and EdU proliferation assays (h). Transfected cells were analyzed by EdU proliferation assays (n=3) (i).


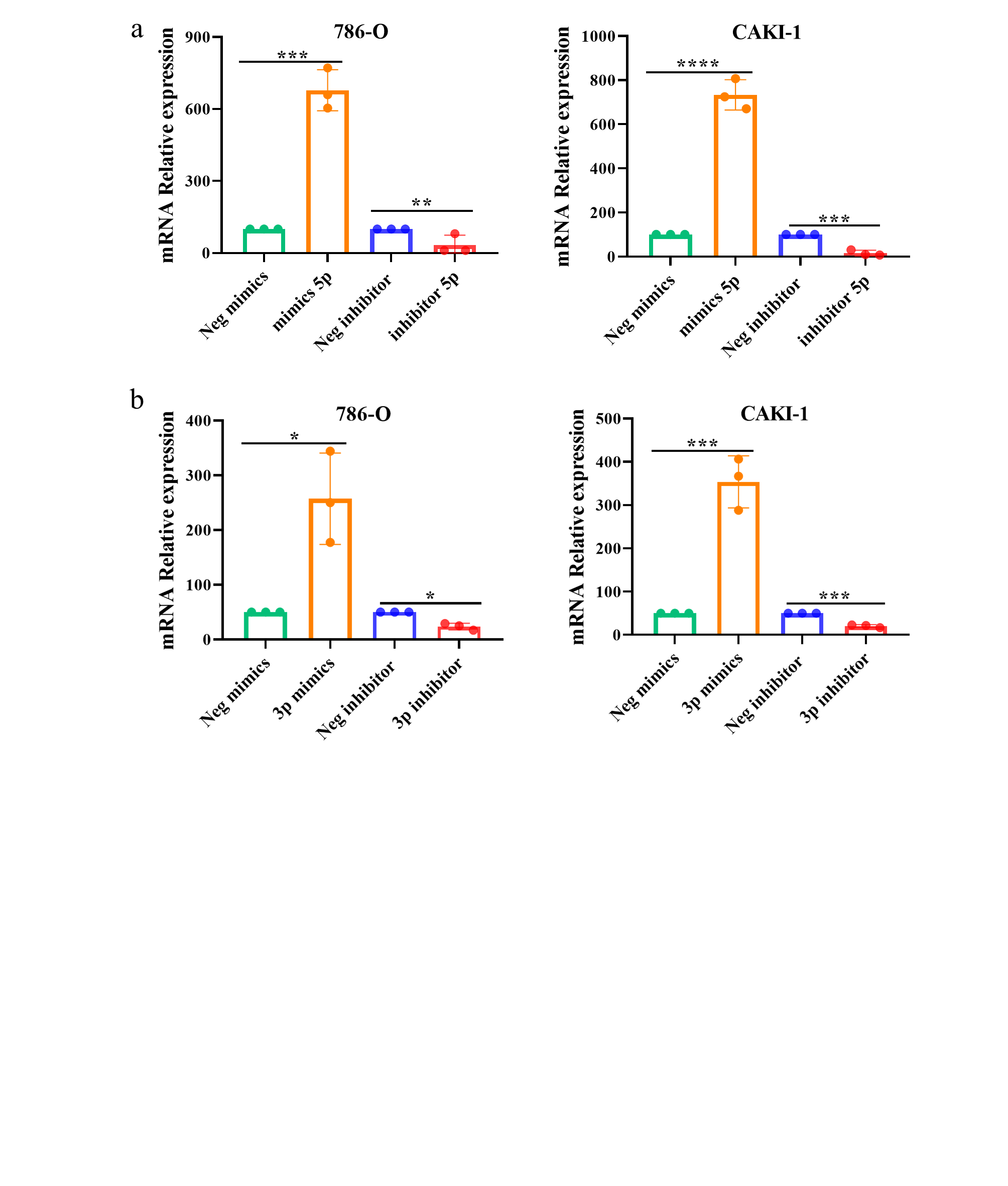


**Supplementary Figure S2. Increase and decrease in the amount of miR-138-5p in ccRCC cells transfected with miR-138-5p mimics and its inhibitor, respectively.** (a) 786-O (left panels) and CAKI-1 (right panels) cells were transfected as indicated. Expression levels of miR-138-5p were examined by qPCR (n=3). (b) 786-O (left panels) and CAKI-1 (right panels) cells were transfected as indicated. Expression levels of miR-138-2-3p were examined by qPCR (n=3).


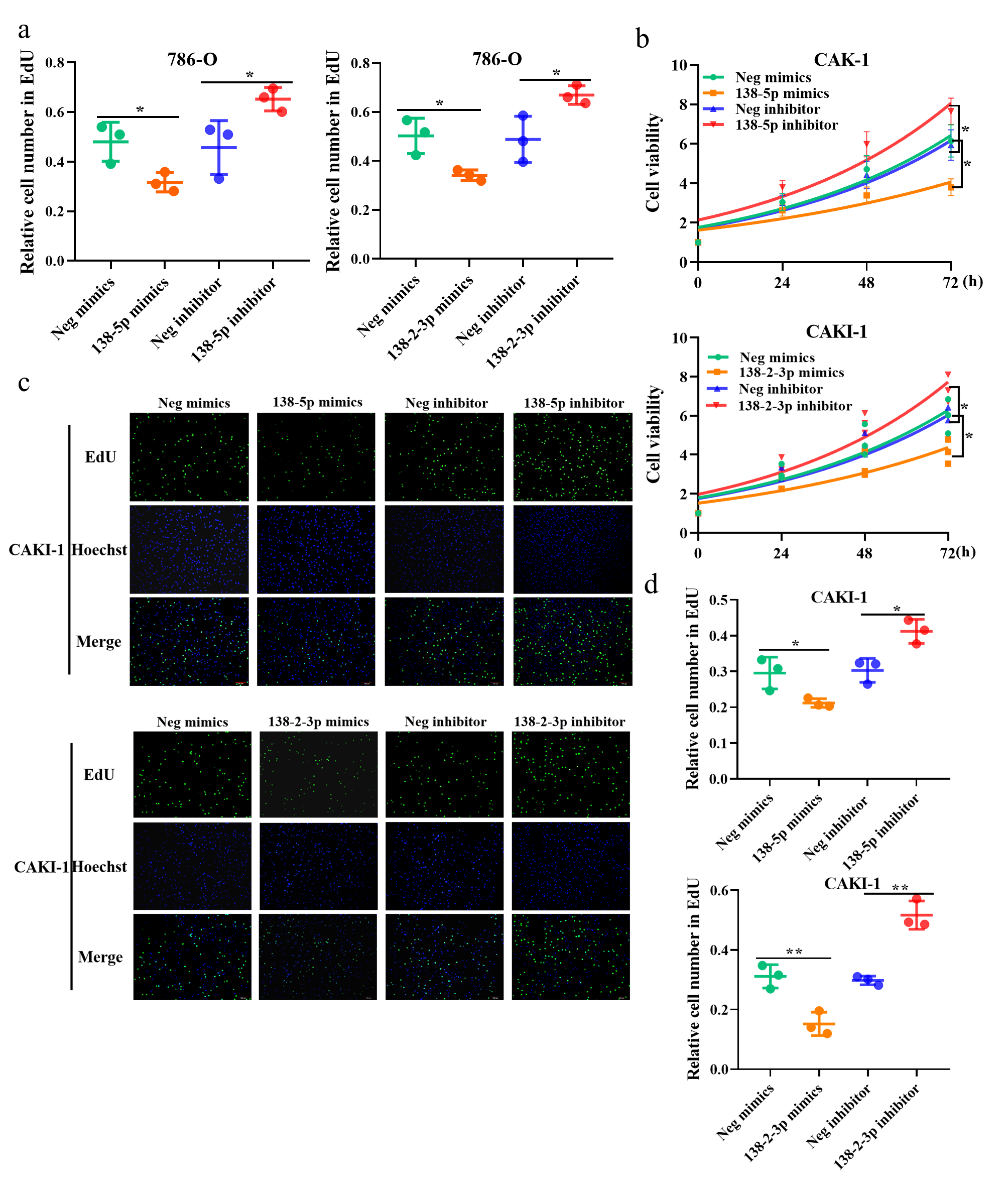


**Supplementary Figure S3. miR-138-5p and miR-138-2-3p suppress proliferation and migration of ccRCC cells.** (a) Proliferation assays. 786-O cells were transfected with the indicated materials. Transfected cells were analyzed by EdU proliferation assays (n=3). (b) CCK-8 assay. CAK-1 cells were transfected with the indicated materials. At the indicated time points after transfection, cells were processed for CCK-8 proliferation assay (n=3). (c and d) Proliferation assays. CAK-1 cells were transfected with the indicated materials. After transfection, cells were subjected to EdU proliferation assay followed by Hoechst staining. The representative pictures were shown (c) and number of EdU-positive cells were scored (n=3) (d).


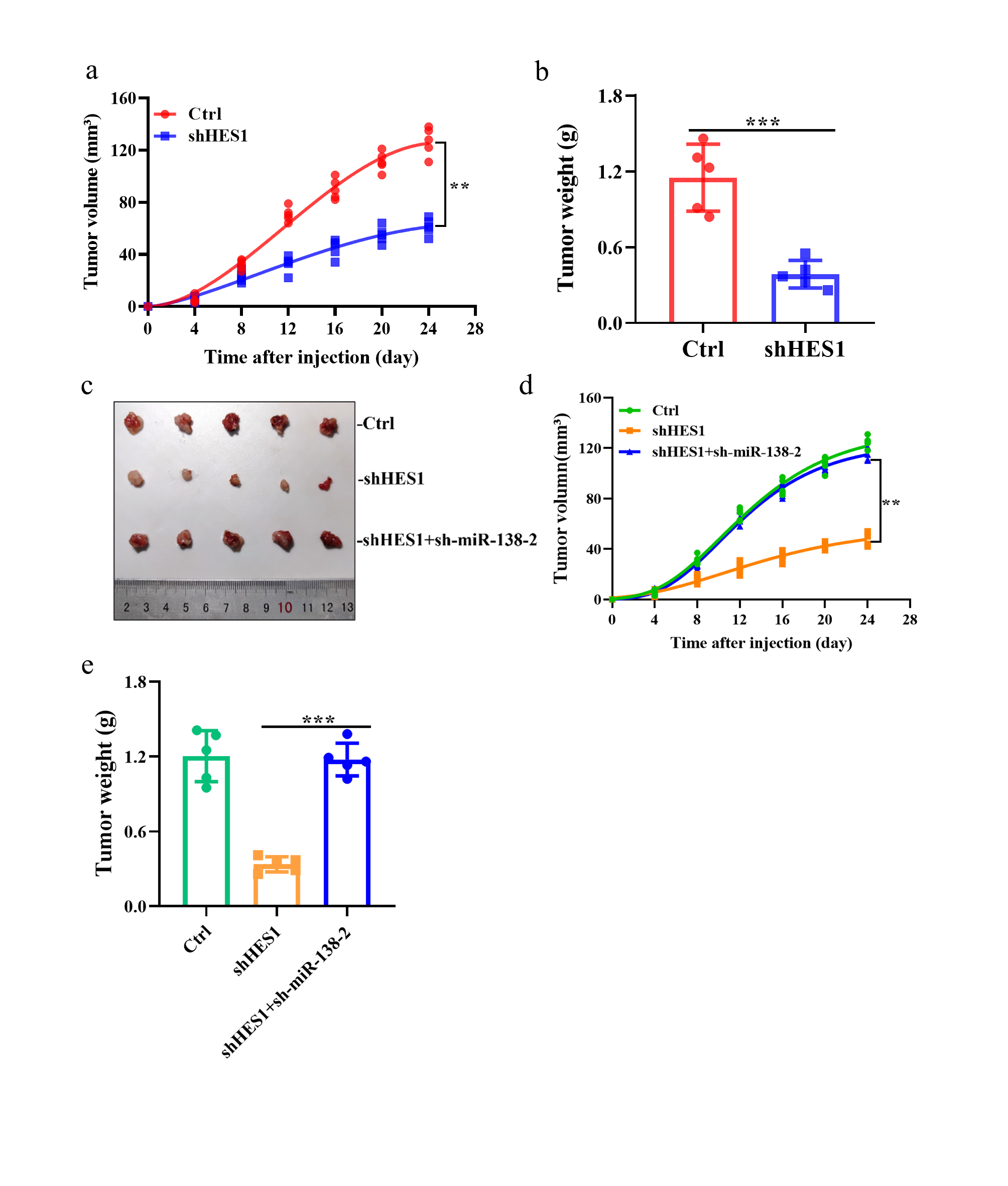


**Supplementary Figure S4. Depletion of HES1 attenuates tumor formation in vivo.**

Nude mice were injected with the control CAK-1 cells or with HES1-knockdown CAK-1 cells. At the indicated time points after injection, tumor volume was measured (n=5) (a). Finally, mice bearing tumors were sacrificed and their tumor weight was scored (n=5) (b). (c-e) Mouse Xenograft. Nude mice were injected with 786-O cells stably overexpressing HES1 with or without miR-138-2 overexpression. The representative tumors were shown (c). Tumor volume (d) and weight (e) were also calculated (n=5).


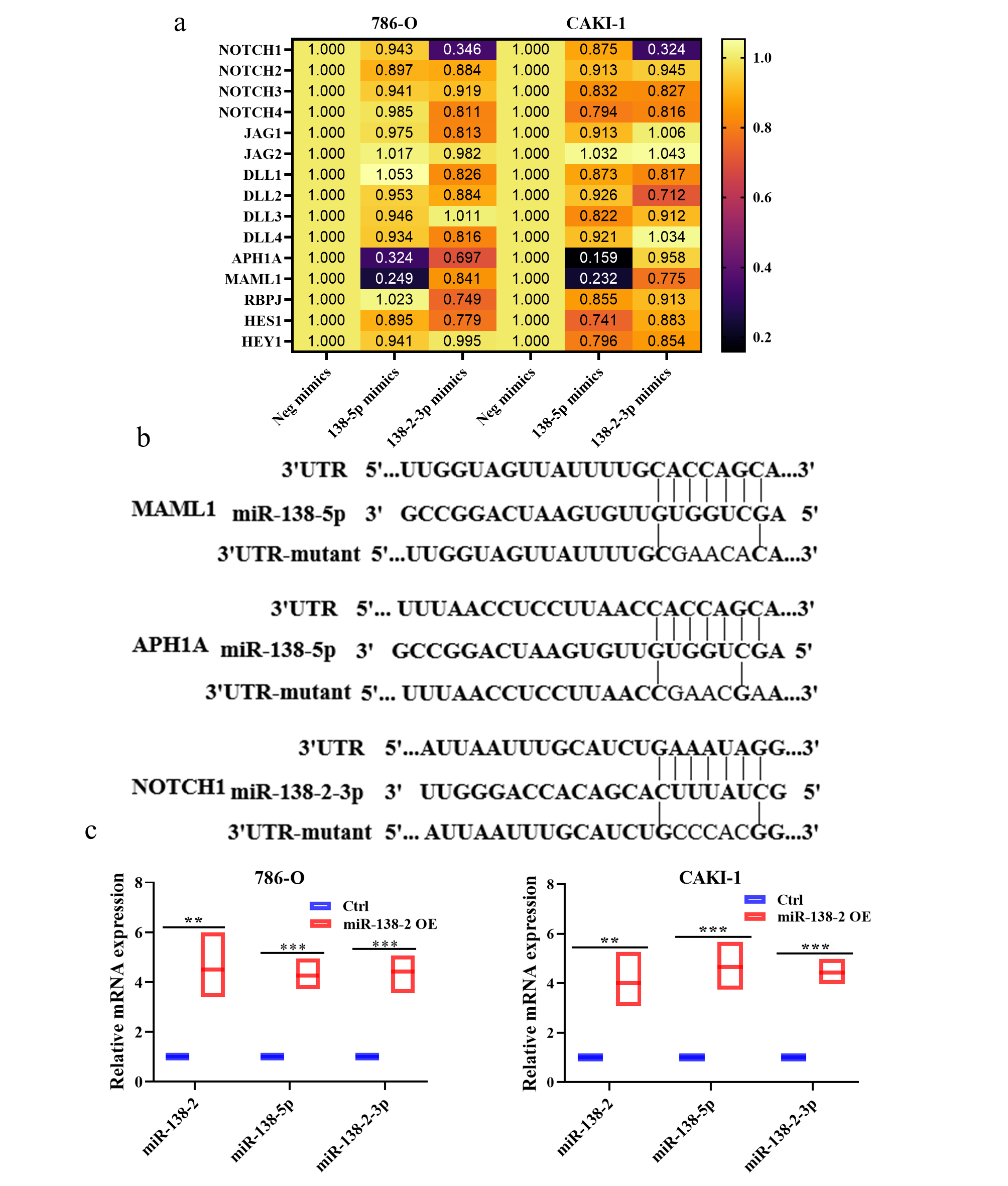


**Supplementary Figure S5. miR-138-2p directly regulates MAML1, APH1A and NOTCH1 in ccRCC cells.** (a) qPCR. 786-O and CAKI-1 cells were transfected with miR-138-5p mimics, miR-138-2-3p mimics or with their corresponding negative controls. Then the whole NOTCH signal genes were analyzed by qPCR assay. (b) Luciferase reporter constructs. Wild-type and the mutated 3’-UTR of MAML1 (top), APH1A (middle) and Notch1(bottom) were shown. (c) miR-138-2 expression plasmid was transfected into 786-O (left panels) or CAK-1(right panels) cells. After transfection, total RNA was prepared and analyzed for pre-miR-138-2, miR-138-5p and miR-138-2-3p by qPCR (n=3).


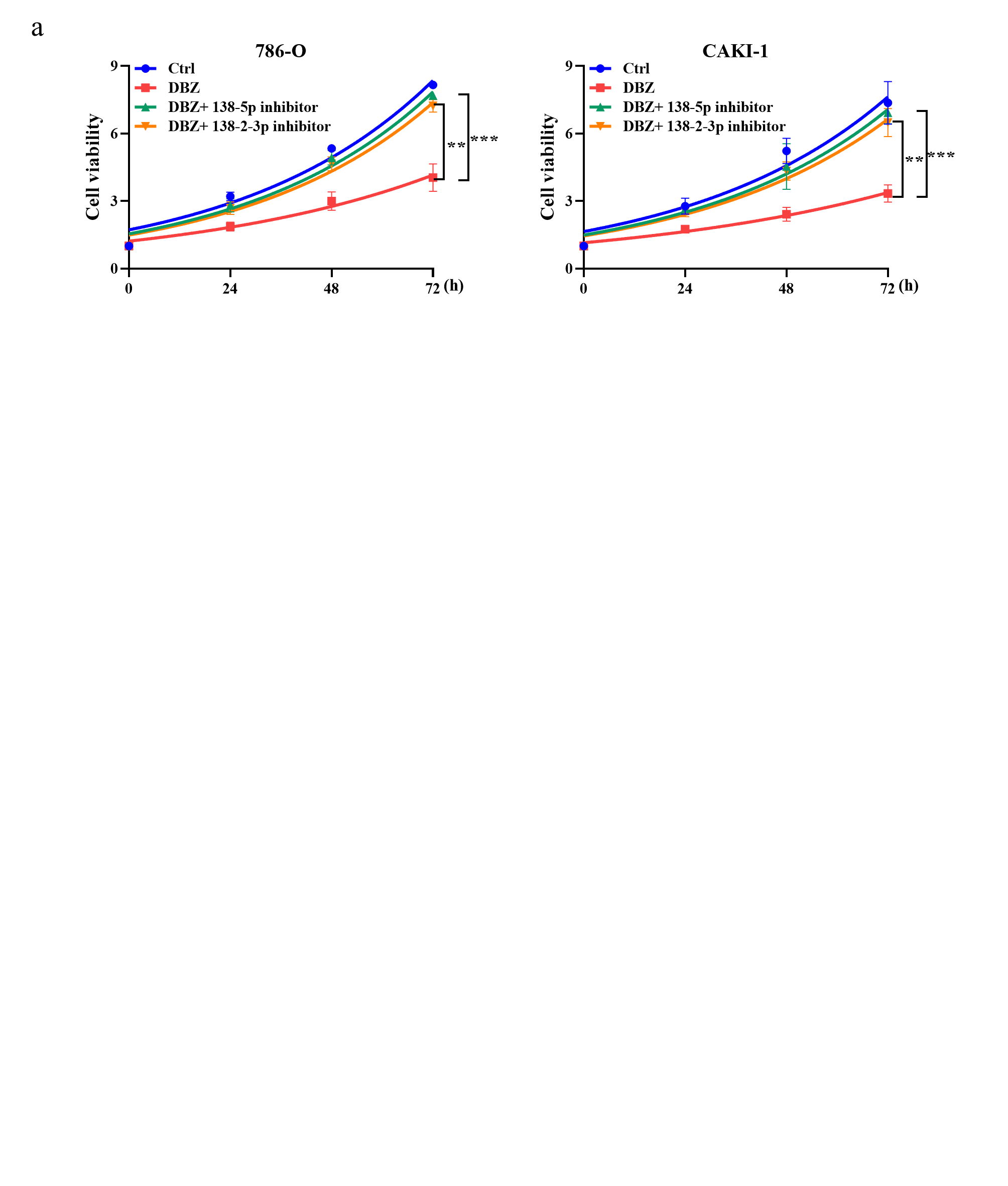


**Supplementary Figure S6. Inhibition of miR-138-5p or miR-138-2-3p could attenuate the effect of DBZ on the activity of ccRCC cells.** (a) CCK-8 assay. 786-O and CAKI-1 cells were transfected with miR-138-5p inhibitor or miR-138-2-3p inhibitors. After transfection, cells were treated with or without DBZ (5 μM/ml). At the indicated time periods after treatment, cell viability was assessed by CCK-8 assay (n=3).
